# Functional and Cognitive Impairments Increased Risks of Outcomes of Healthcare Utilization in Patients With Stroke Receiving Home and Community-Based Care in Taiwan

**DOI:** 10.3389/fpubh.2021.644911

**Published:** 2021-08-05

**Authors:** Li-Fan Liu, Wei-Ming Wang, Jung-Der Wang

**Affiliations:** ^1^Institute of Gerontology, College of Medicine, National Cheng Kung University, Tainan, Taiwan; ^2^Department of Statistics, College of Management, National Cheng Kung University, Tainan, Taiwan; ^3^Department of Public Health, College of Medicine, National Cheng Kung University, Tainan, Taiwan; ^4^Department of Occupational and Environmental Medicine, National Cheng Kung University Hospital, College of Medicine, National Cheng Kung University, Tainan, Taiwan

**Keywords:** long-term care, mortality, rehospitalization, re-emergency, stroke survivors

## Abstract

**Aim:** Stroke is a leading cause of disability; however, little is known about the outcomes of the utilization of long-term care (LTC) recipients in Taiwan. This study aimed to quantify the burdens of disease of stroke survivors receiving LTC by evaluating the outcomes of their utilization including mortality, readmissions, and re-emergency within 1 year after diagnoses of strokes.

**Methods:** By interlinkages among the national mortality registry, LTC dataset (LTC-CM), and the National Health Insurance Research Dataset (NHIRD), the outcomes and the factors associated with receiving LTC up to 1 year were explored. Patients were aged 50 years and over with an inpatient claim of the first diagnosis of stroke of intracerebral hemorrhage (ICH) and ischemic stroke during 2011–2016. Outcomes of the healthcare utilization include rehospitalization and re-emergency.

**Results:** There were 15,662 patients with stroke who utilized the LTC services in the dataset among the stroke population in NHIRD. Stroke survivors receiving LTC showed no difference in clinical characteristics and their expected years of life loss (EYLL = 7.4 years) among those encountered in NHIRD. The LTC recipients showed high possibilities to be rehospitalized and resent to emergency service within 1 year after diagnosis. Apart from the comorbidity and stroke severity, both the physical and mental functional disabilities and caregiving resources predicted the outcomes of the utilization.

**Conclusions:** For stroke survivors, both severe functional impairments and cognitive impairments were found as important factors for healthcare utilizations. These results regarding reserving functional abilities deserve our consideration in making the decision on the ongoing LTC policy reform in the aged society of Taiwan.

## Introduction

Stroke is a leading cause of disability and morbidity associated with increased economic burden related to acute treatment, poststroke care (PSC), and rehabilitation. Although notable therapeutic advances have contributed to reducing brain damage and disability in patients with stroke (1), many of them are still left with a functional impairment that prevents them from performing basic activities of daily living. Thus, stroke survivors would still face challenges of long-term care (LTC) and continued rehabilitation, which usually result in a tremendous burden.

The Taiwan government launched the LTC 1.0 policy in 2007, which aimed at assisting frail elderly people with LTC needs. The initial objective was to develop a system of home and community-based services (HCBS), including home services, adult day care, home nursing care, home and community-based rehabilitation, home meal delivery, palliative care for caregivers, and transportation services. To facilitate service delivery, the government began to transform the LTC 1.0 into a new reform of the LTC 2.0 policy system in 2016 (2). Under LTC 2.0 policy, more transitional care linking with LTC has been provided, such as planning for home discharge in the hospitals and home healthcare. However, little has been evaluated about the outcomes of the utilization of LTC recipients under the LTC 1.0 policy. By interlinkages among the national mortality registry, LTC dataset (LTC-CM), and the National Health Insurance Research Dataset (NHIRD), this study aimed to quantify the burdens of disease of patients with different subtypes of strokes under LTC by evaluating their mortality and outcomes of the utilization including readmissions and re-emergency within 1 year after the diagnoses of strokes under the National Health Insurance (NHI). The results of these real-world data could provide additional evidence for improving service delivery and reform of the LTC policy 2.0 in Taiwan.

### Literature Review

The risk of stroke increases with age, is more common among women, and is a major contributor to long-term disability, especially among the elderly (3). Previous studies in the US indicated that nearly 90% of strokes are ischemic, which are caused by a blockage in the cerebral artery, which restricts blood flow (4). Based on the International Classification of Diseases, Ninth Revision, Clinical Modification (ICD-9-CM), the incidence of stroke admissions was high among the acute ischemic stroke (AIS) and intracerebral hemorrhage (ICH).

Caring for stroke survivors imposes a substantial economic burden on society (3). The activities of daily living (basic and instrumental) are reliable indicators of the functional status of patients affected by stroke for the initial evaluation (5). An Australian study showed that among 3-month stroke survivors, 74% required assistance with activities of daily living and received informal care from family or friends (6). In the U.S., 75 % of survivors returned home 1 year after index stroke admission (7). In Taiwan, an earlier study showed that about 15.2% were institutionalized after 6 months of stroke (8).

Stroke survivors also suffered from the heavy burden of disease. In the US, stroke is one of the top ten contributors to Medicare costs (4). Taiwan has had a well-established NHI system with over a 99% coverage rate for acute care since 1995. It is estimated that in Taiwan, nearly 75% of those who need LTC are aged 65 years and over (9). Although the recipients of such LTC program must be reassessed every 6 months under the regulations of 1.0 LTC policy, to the best of our knowledge, the outcomes of the utilization have not been systematically evaluated for continued quality assurance, improvement of the care plan, and possible development into an insurance system.

Therefore, from the perspective of LTC, this study explored the profile of stroke survivors who utilized formal HCBS in Taiwan and examined their 1-year outcomes and the predicting factors after index stroke admission and receiving LTC. In this study, we summarized the profile (sociodemographics and functional disabilities) of the LTC recipients with stroke during 2011–2016 among those encountered in NHI of Taiwan, including their life expectancy (LE) and expected years of life loss (EYLL). Then, LTC recipients were enrolled for the analyses of the outcomes related to healthcare utilizations, such as rehospitalization and re-emergency up, to 1 year after index stroke admission and the associated predicting factors. With evidence coming from these real-world data, we hope to add information regarding health outcomes of stroke care in HCBS and hope to be useful to sustain stroke care under the LTC system in Taiwan.

## Materials and Methods

The study protocol (no: A-ER-106-183) was approved by the Institution Review Board before commencement, and no conflicts of interest exist between the authors and the goals of this study.

### Long-Term Care Data Set

The data were obtained from the national LTC-CM in Taiwan. This dataset has been maintained by the LTC center in each county, in which the health and functional indicators of care recipients are recorded in an initial needs assessment and are reassessed by care managers during the follow-up process (10, 11). This study is designed to interlink records of the LTC-CM during 2011–2016 with NHIRD and to explore the outcome of healthcare utilization and the factors associated with receiving HCBS up to 1 year.

### Case Selection

**Figure 1:**
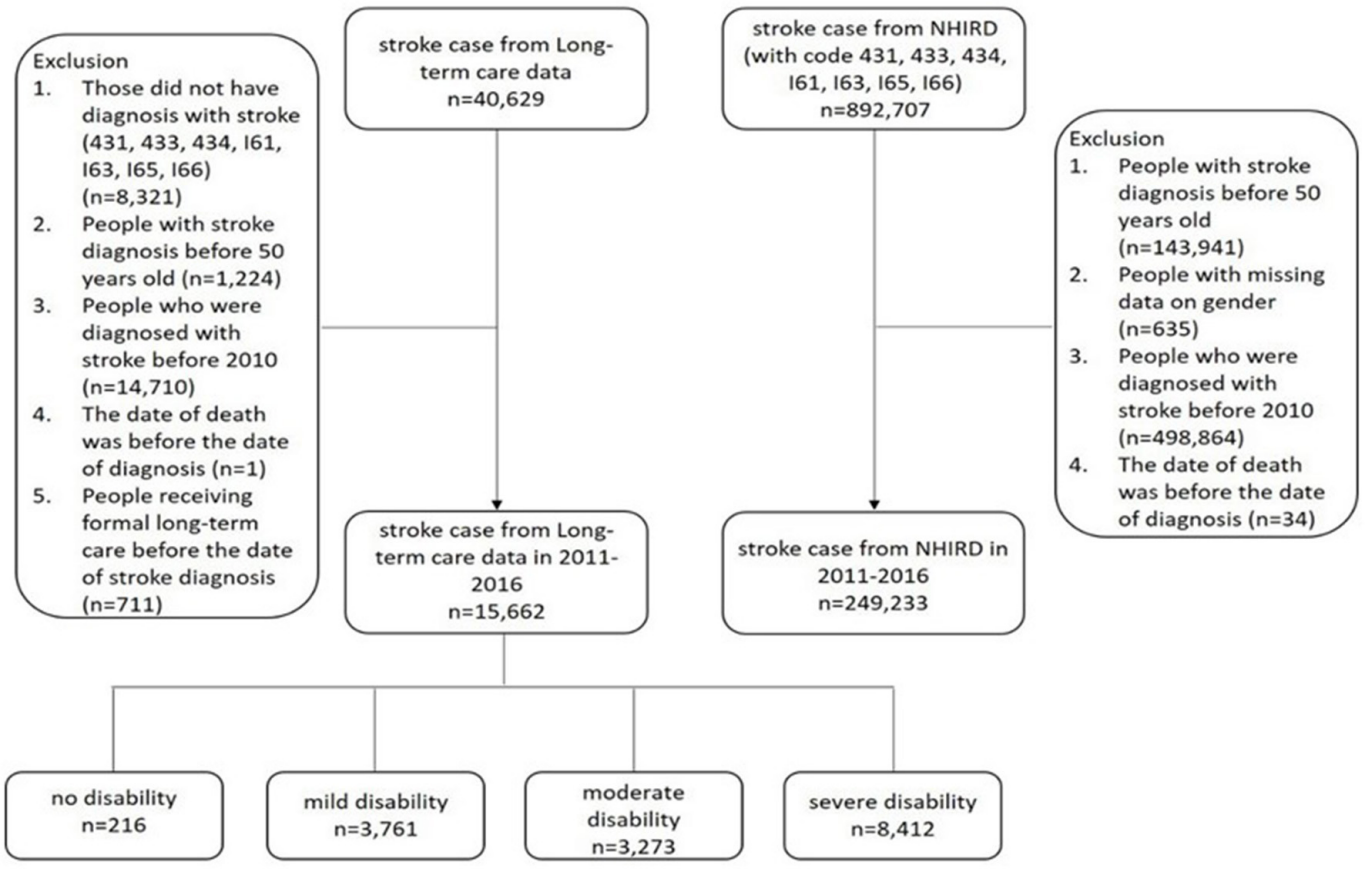
Flow chart of the study samples.

The inclusion criteria were patients aged 50 years and over with an inpatient claim of the first diagnosis of stroke defined as follows: confirming principal diagnosis codes for primary or secondary ICD-9 codes of ICH (431.x) and ischemic stroke (433.x and 434.x) from January 2011 to December 2016, in any position by ICD-9-CM and its matched codes in ICD-10 since 2016. The flowchart of the study population is shown in Figure 1.

To identify the LTC recipients with diagnoses of strokes mentioned above, cases were further identified by linking the LTC dataset with NHIRD. In total, there were 249,233 cases identified, and among them 15,662 persons received HCBS in the LTC system of Taiwan after discharge from hospitals.

### Statistical Analysis

For the analyses of the predictors of healthcare utilizations (rehospitalization and re-emergency) up to 1 year, the negative binomial regression models were constructed and the dependent variables are a count of events by controlling the time periods staying in the HCBS system. The analyses were performed using Statistical Analysis System (SAS) version 9.4.

After verifying the survival status of all cases through cross-referencing with the national mortality registry at the end of the follow-up period (December 31, 2016), the Kaplan-Meier method was used to estimate the survival function based on the 2011–2016 follow-up data. Extrapolation of survival to lifetime for the stroke cohort after the termination of the follow-up period was conducted. Because the LE is the summation of the area under the lifetime survival curve, we would be able to obtain the EYLL by comparing the LEs between the stroke cohort and that of the correspondingly matched referents (12, 13). The approach was mathematically proved valid for predicting the LE under the assumption of constant excess hazard earlier (14–16). Moreover, the software has been updated in the iSQoL 2 with a new algorithm of “rolling over” month by month and only assumes constant hazard within the extrapolated 1 month to accommodate diseases with versatile courses, such as stroke (12).

### Measures of Stroke Severity and Comorbidities

In this study, stroke severity index (SSI) and modified Charlson comorbidity index (modified CCI) were used to identify stroke severity and comorbidities. A claims-based SSI is found to be a valid proxy for the National Institutes of Health Stroke Scale (NIHSS) and an effective adjustment for stroke severity in studies of AIS or ICH outcome with administrative claims data (17–19).

The severity of comorbidities was summarized using a modified version of the CCI that excluded cerebrovascular disease and hemiplegia (20). The modified CCI was dichotomized into low comorbidity ( ≤ 1) or high comorbidity (>1) for analyses.

## Results

There were 15,662 patients with stroke who utilized the HCBS after the selection process of patients. The detailed profiles of the LTC recipients with stroke from 2011 to 2016 are summarized in Table 1. It showed that the average age of women was higher than men. The ratio of ischemic stroke vs. hemorrhagic stroke was slightly higher than 4:1, and the mean age of those with a higher SSI was younger. More than half (53.1%) of them entered into the LTC system <6 months after stroke admission. Approximately 19.1% of the LTC recipients were from mid-low/low-income households, and more than 90% of them were accompanied by caregivers at own home at the first entry to LTC.

**Table 1:** The profile (sociodemographics and functional disabilities) of the long-term care (LTC) recipients with stroke from 2011 to 2016.

	***N* (%)**	**Age**	***p*-value**
Age (years)	15,662 (100%)	73.7 ± 9.4	
**Gender**
Male	8,702 (55.6%)	72.0 ± 9.7	<0.001
Female	6,960 (44.4%)	75.1 ± 8.6	
**Stroke subtype**
Hemorrhagic Stroke	2,921 (18.6%)	69.3 ± 10.3	<0.001
Ischemic Stroke	12,741 (81.4%)	74.3 ± 8.9	
**Stroke severity index**
Mild ≤ 5	5,611 (35.8%)	73.6 ± 8.8	<0.001
Moderate 5–13	4,660 (29.8%)	74.0 ± 9.1	
Severe >13	5,391 (34.4%)	72.5 ± 10.1	
**Time to long-term care**
0–6	8,311 (53.1%)	75.5 ± 8.4	<0.001
6–12	2,244 (14.3%)	71.2 ± 10.1	
12–24	2,234 (14.3%)	71.0 ± 10.1	
24+	2,873 (18.3%)	70.8 ± 9.5	
**Welfare status**
Non-low-income households	12,673 (80.9%)	74.7 ± 8.7	<0.001
Mid-low/low incomes households	2,989 (19.1%)	67.8 ± 10.2	
**Caregivers**
Yes	14,403 (92.0%)	73.4 ± 9.4	0.005
No	1,259 (8.0%)	72.6 ± 9.6	
**ADL^†^ difficulties**
Severe (score 0–30)	8,412 (53.7%)	75.2 ± 8.9	<0.001
Moderate (score 31–60)	3,273 (20.9%)	72.8 ± 9.4	
Mild (score 61–100)	3,977 (25.4%)	70.7 ± 9.5	
**IADL^‡^ difficulties**
Severe (score <8)	10,010 (63.9%)	74.3 ± 9.1	<0.001
Mild (score ≥ 8)	5,652 (36.1%)	71.7 ± 9.6	
**CESD^§^**
No depressive symptoms	10,684 (68.2%)	72.5 ± 9.4	<0.001
Depressive	4,978 (31.8%)	75.2 ± 9.1	
**MDAI^¶^ cognition**
Severe impairment	5,446 (34.8%)	75.8 ± 8.9	<0.001
Moderate impairment	2,399 (15.3%)	74.7 ± 8.7	
Mild impairment	1,972 (12.6%)	73.4 ± 9.1	
No impairment	5,848 (37.3%)	70.5 ± 9.4	

† *ADL, activity of daily living*.

‡ *IADL, Instrumental activity of daily living*.

§ *CESD, Center for Epidemiologic Studies Depression, the thresholds of depressive tendency: male ≥12, female ≥10*.

¶ *MDAI, multi-dimensional assessment instrument for cognition*.

In terms of self-care functions, more than half of them (53.7%) were suffering from severe difficulties in activities of daily living (ADLs <30). About 63.9% of the LTC recipients showed a score of instrumental activities of daily living (IADL) lower than 8. Based on the thresholds of the Center for Epidemiologic Studies Depression (CESD), 31.8% of them seemed depressive and about 34.8% of the LTC recipients showed severe cognitive impairment (Table 1).

Table 2 shows the clinical characteristics of stroke patients receiving LTC among those encountered in NHI of Taiwan. The mean age of the LTC recipients with stroke was significantly older than their counterparts in NHIRD [73.7 ± 9.4 vs. 70.7 ± 10.6, standardized difference (SD) = 0.3]. Alternatively, all the calculated SDs of comorbidities and severity between patients with stroke receiving LTC and those encountered in the NHI were below 0.2. For example, the two groups looked similar. Although the LE of those receiving LTC was 1.96 years lower than that of the NHI patients with stroke, there was no difference in their EYLL, or both cohorts lost about 7.4 years of LE. It indicates that the difference of their LE would be from a different age of onset.

**Table 2:** The prevalence and means of clinical characteristics *at baseline* of patients with stroke receiving LTC among those hospitalized through National Health Insurance (NHI) of Taiwan during 2011–2016.

**Clinical characteristic**	**LTC recipients (*n* = 15,662)**	**NHI (*n* = 249,233)**	**SMD**
Age (years), Mean (SD)	73.7 ± 9.4	70.7 ± 10.6	0.30^*^
Sex (Female), *N* (%)	6,960 (44.4)	103,703 (41.6)	0.06
Stroke subtype			
Hemorrhagic, *N* (%)	2,921 (18.6)	43,962 (17.6)	0.03
Ischemic, *N* (%)	12,741 (81.4)	205,271 (82.4)	
Modified CCI^†^			
0 or 1, *N* (%)	9,885 (63.1)	157,050 (63.0)	<0.01
>1, *N* (%)	5,777 (36.9)	92,183 (37.0)	
Hypertension, *N* (%)	12,212 (78.0)	185,410 (74.4)	0.08
Diabetes mellitus, *N* (%)	6,287 (40.1)	94,626 (38.0)	0.04
Hyperlipidemia, *N* (%)	5,300 (33.8)	89,668 (36.0)	0.04
Prior stroke, *N* (%)	3,518 (22.5)	64,392 (25.8)	0.08
Atrial fibrillation, *N* (%)	2,324 (14.8)	28,563 (11.5)	0.10
Coronary heart disease, *N* (%)	1,689 (10.8)	26,952 (10.8)	<0.01
Chronic kidney disease, *N* (%)	1,312 (8.4)	24,393 (9.8)	0.05
SSI^‡^ score	10.2 ± 6.2	9.1 ± 6.2	0.18
Hemorrhagic Stroke	15.6 ± 5.2	14.8 ± 5.9	0.14
Ischemic stroke	9.0 ± 5.7	7.9 ± 5.6	0.19
**Life expectancy (in year)**	6.12 ± 0.12	8.08 ± 0.10	17.74^*^
**Expected years of life loss (in year)**	7.38 ± 0.14	7.36 ± 0.10	0.17

† *CCI, Charlson comorbidity index*.

‡ *SSI, stroke severity index*.

* *SMD, standardized mean difference is usually considered statistically significant if >0.2*.

Table 3 summarizes the 1-year mortality, rehospitalization, and re-emergency among the LTC recipients. We found that the mortality rate of the LTC cohort seemed to be generally low (8.4%) during the first year. However, the proportion of rehospitalization seemed high. It showed the proportion of about 30.7% within 1 month and 63.2% within 1 year among LTC recipients. The 1-year average times of rehospitalization were also significantly higher than the nationwide cohort (1.69 ± 2.21 vs. 1.02 ± 1.76), especially among those with hemorrhagic stroke. The proportion of re-emergency within 1 month appeared similar between the two cohorts (14.6 vs. 12.8%), the difference increased gradually along time, and the average frequency of re-emergency at the end of 1 year for LTC recipients was significantly higher than that of the nationwide cohort (1.65 ± 2.46 vs. 1.01 ± 2.09).

**Table 3:** The mortality and follow-up indices (rehospitalization and re-emergency) of patients with stroke receiving LTC among the National Health Insurance Research Dataset (NHIRD) in Taiwan during 2011–2016.

**Characteristic**	**LTC recipients (*n* = 15,662) *N* (%)**	**NHIRD (*n* = 249,233) *N* (%)**	***p*-value**	**SMD**
Age (years)	73.7 ± 9.4	70.7 ± 10.6		0.30^*^
**Mortality**
<1 month	44 (0.3)	21,611 (8.7)	<0.001	0.41^*^
1–3 months	238 (1.5)	9,976 (4.0)	<0.001	0.15
3–6 months	365 (2.3)	7,290 (2.9)	<0.001	0.04
6–12 months	673 (4.3)	9,833 (4.0)	<0.001	0.02
Total (0–12 months)	1,320 (8.4)	48,710 (19.5)	<0.001	0.02
**Re-hosp**.
<1 month	4,795 (30.7)	36,359 (15.9)	<0.001	0.35^*^
1–3 months	4,812 (30.7)	48,268 (19.4)	<0.001	0.26^*^
3–6 months	4,413 (28.2)	42,108 (16.9)	<0.001	0.27^*^
6–12 months	4,897 (31.3)	48,547 (19.5)	<0.001	0.27^*^
Total (0–12 months)	9,902 (63.2)	110,182 (44.2)	<0.001	0.39^*^
1-year average	1.69 ± 2.21	1.02 ± 1.76		0.34^*^
Hemorrhagic Stroke	2.21 ± 2.68	1.19 ± 2.08	<0.001	0.43^*^
Ischemic stroke	1.58 ± 2.07	0.98 ± 1.68	<0.001	0.32^*^
**Re-ER**
<1 month	2,276 (14.6)	29,040 (12.8)	<0.001	0.05
1–3 months	3,802 (24.3)	39,737 (15.9)	<0.001	0.21^*^
3–6 months	4,034 (25.8)	40,649 (16.3)	<0.001	0.23^*^
6–12 months	5,497 (35.1)	54,736 (22.0)	<0.001	0.29^*^
Total (0–12 months)	9,067 (57.9)	109,145 (43.8)	<0.001	0.28^*^
1-year average	1.65 ± 2.46	1.01 ± 2.09	<0.001	0.28^*^
Hemorrhagic Stroke	1.54 ± 2.32	0.80 ± 1.73	<0.001	0.36^*^
Ischemic stroke	1.67 ± 2.48	1.06 ± 2.16	<0.001	0.26^*^

* *Standardized mean difference (SMD) is usually considered statistically significant if >0.2*.

Table 4 presents the inferential analyses for predictors of rehospitalization and re-emergency up to 1 year among the LTC recipients with stroke (*n* = 12,789). Based on the construction of negative binomial regression models, we found that men, high severity, more comorbidity, and those without caregivers were more likely to be readmitted, even after adjustment for multiple functional disabilities. Functional disabilities including ADLs, IADLs, and cognitive impairments were significant predictors for rehospitalization in the follow-up of 1 year. For the re-emergency rates (Table 4), the results again showed that functional disabilities including severe dependence on ADLs, CESD, and severe cognitive impairments were significant predictors for re-emergency in the follow-up of 1 year. That is, subjects with severe functional disabilities of self-care, daily activities, cognition, and depression would be more likely to be rehospitalized or sent to the emergency by the end of the follow-up of 1 year.

**Table 4:** Estimates of incidence rate ratio (IRR) with 95% CI for risk factors of 1-year outcomes (rehospitalization, re-emergency) among the LTC recipients with stroke based on negative binomial regressions.

	**Times of Re-hospitalization in 1 year**	**Times of Re-emergency in 1 year**
**Risk factors**	**IRR (95% CI)**	**IRR (95% CI)**
Age	0.98 (0.98, 0.98)^***^	1.01 (1.00, 1.01)^***^
Female (vs. Male)	0.81 (0.78, 0.81)^***^	0.85 (0.81, 0.89)^***^
Mid/low vs. non-low incomes	0.92 (0.87, 0.97)^**^	1.00 (0.94, 1.06)
No Caregivers (vs. with caregivers)	1.14 (1.06, 1.24)^***^	1.16 (1.06, 1.26)^***^
Hemorrhagic Stroke (vs. Ischemic Stroke)	1.01 (0.96, 1.07)	0.95 (0.89, 1.01)
Charlson comorbidity index	1.05 (1.04, 1.07)^***^	1.09 (1.08, 1.11)^***^
Stroke severity index	1.03 (1.03, 1.03)^***^	1.00 (0.99, 1.00)
**Functional disability**		
ADL^†^ severe (vs. ADL^†^ mild)	1.54 (1.45, 1.65)^***^	1.47 (1.36, 1.57)^***^
ADL moderate^†^ (vs. ADL^†^ mild)	1.24 (1.16, 1.32)^***^	1.07 (0.99, 1.15)
IADL severe (vs. IADL^‡^ mild)	1.05 (1.00, 1.10)^*^	1.05 (0.99, 1.10)
CESD^§^ depression (vs. no depression)	1.02 (0.97, 1.08)	1.08 (1.02, 1.14)^**^
Cognition impairment mild (vs. no imp.)	0.98 (0.92, 1.05)	1.05 (0.98, 1.13)
Cognition impairment moderate (vs. no. imp.)	1.02 (0.96, 1.09)	1.07 (0.99, 1.15)
Cognition impairment severe (vs. no imp.)	1.12 (1.05, 1.20)^***^	1.19 (1.11, 1.28)^***^

† *ADL, activity of daily living*.

‡ *IADL, Instrumental activity of daily living*.

§ *CESD, Center for Epidemiologic Studies Depression, the thresholds of depressive tendency: male ≥12, female ≥10*.

* *p <0.05*,

** *p <0.01, and*

*** *p <0.001*.

## Discussions

Stroke is a major contributor to long-term functional disability, especially among the elderly (3, 21). For stroke survivors, the need for LTC seems inevitable. The study explored the profile of stroke survivors who utilized formal HCBS of Taiwan and followed their utilization outcomes up to 1 year. The influencing factors of utilization outcomes after index stroke admission were also examined.

The LTC policy in Taiwan has been launched by the central government since 2007 aiming at developing the HCBS. For stroke survivors, apart from institutional care, home nursing and home services remained the primary LTC services during the time period of the study. However, among stroke survivors with LTC needs, how many received the help from the HCBS and how were their healthcare utilization outcomes and predictors have not yet been explored and somehow ignored due to the integrating gap between acute care and LTC. Although the post-acute care plan has been launched in a few local hospitals during the follow-up time since 2014, the outcomes of the utilization of stroke survivors receiving the HCBS afterward and the predictors would be important to effort improvement on clinical interventions and the ongoing LTC policymaking. To the best of our knowledge, the outcomes of the utilization of stroke survivors in receiving the HCBS up to 1 year and the predictors were first analyzed in Taiwan.

Previous studies showed that <9% of patients with AIS received long-term home care after hospitalization in the US (21). Our results found that for the patients with ICH and AIS, there was about 6.3% of the stroke survivors who used the HCBS after index stroke admission during the study period. The utilization rate could be influenced by many contextual factors in various countries. In Taiwan, the development of LTC infrastructure and lack of workforce may cause the low utilization rates during the study period. Some other factors from the demand side may be also influential, such as in Taiwan, preference of families of hiring foreign care workers at own homes to support informal caregiving instead of using formal HCBS subsidized by the central government (10, 22).

In general, we found no difference between stroke survivors at baseline among who utilized LTC or not after index stroke admission in NHIRD. Regarding LE and EYLL, the LTC recipients were found to be those with shorter LE that may be partly explained by their higher mean age. However, there were no differences in EYLL between the two groups in long term.

For the mortality rates, the result showed that the LTC recipients were those stroke survivors with lower percentages of mortality, especially within 1 month of occurring stroke. The mortality rates among the LTC recipients had increased gradually after index stroke admission, however, showing no statistical difference with their counterparts nationwide after 1 month. Therefore, it seems not surprised that those who utilized LTC or not showed no influence in terms of EYLL and mortality.

Regarding the utilizations, all-cause readmission rates and re-emergency rates within 1 year of utilizations of HCBS had been focused on and analyzed in this study. The higher rehospitalization and re-emergency rates and the 1-year average of utilizations among the LTC recipients were, however, significantly higher than their counterparts nationwide. A previous study on poststroke in Singapore showed that the highest utilization occurred in the first quarter poststroke across all service types and decreased with time since stroke (23). This study found that the percentage of rehospitalization and re-emergency among patients with stroke has increased within 1-year follow-up. If the results mean worse outcomes or because of higher accessibilities of healthcare utilizations after receiving HCBS was not examined in this study and needs to be further addressed. However, these results deserve our notice and consideration in making the decision on national health and LTC policy, especially on prevention.

Few studies have systematically evaluated predictors of readmission after stroke, a measure of inpatient quality of care (24). The previous study indicated that the use of care after hospitalization for AIS was greatest for individuals with more severe strokes, lower functioning at hospital discharge, older age, unmarried, and black in the US (21). This study has shown some key predictors for outcomes of the utilization up to 1 year after index stroke admission. For the rehospitalization, apart from the sociodemographic factors of the LTC recipients, the modified CCI and SSI predicted the utilization. For the re-emergency, the result showed that hemorrhagic stroke has less incidence rate ratio (IRR) than ischemic stroke for re-emergency in the study. The findings also showed the consistency that the modified CCI was a significant predictor for subsequent rehospitalization and re-emergency within 1-year follow-up.

Moreover, the levels on functional disabilities including both physical (ADLs and IADLs) and mental (CESD and cognition status) were found to be key predictors in the outcomes of the utilization (rehospitalization and re-emergency). The study indicated that functional impairment is associated with increased risk of 30-day, all-cause hospital readmission in Medicare seniors, especially those admitted for heart failure, myocardial infarction, or pneumonia (25). Our study found that the influence exists after the follow-up up to 1 year after index stroke admission. Both the physical and the mental functional impairments were influential for the rehospitalization and re-emergency, especially for those in severe conditions. For senior stroke survivors in Taiwan, functional impairment and severe cognitive impairment were found as important factors in preventing readmissions, and severe functional impairment, depression, and severe cognitive impairment were found as important factors in preventing re-emergency.

Our results reflect the notion proposed by the WHO that the composite of all the physical and mental capacities of an individual at any point in time, or intrinsic capacity, is important. Both the health and social care services should be targeted toward preventing and managing declines in intrinsic capacity and improving functional ability in the elderly (26, 27). For example, LTC would be a means to ensure and facilitate the elderly with a significant loss of capacity for healthy aging through reengineering and reconstructing their functional ability and intrinsic capacity.

As part of the learning process, this study examined the outcomes of services of patients with stroke receiving the HCBS and hoped to be useful to facilitate the policy to provide closer continuity of LTC services with quality.

### Limitations

Some limitations in the study need to be addressed. First, to narrow down stroke survivors, only patients with ICH and AIS with admission index from NHIRD with or without LTC use were identified in the study by linking the LTC dataset. Patients with stroke who were not hospitalized or treated at outpatient clinics were not included in the study. Because patients with a definite diagnosis of stroke can be waived from copayment for the first month in the NHI system of Taiwan, our collection would include almost all patients with stroke except those with minimal transient ischemic attack and would be relatively representative. Second, only the LTC dataset was available with information of functional disabilities of patients and no such data collected in NHIRD. Thus, our regression models could only present those ever-received services from LTC. Finally, we chose to follow up on 1-year outcomes of the healthcare utilization in the study instead of a lifelong perspective. Future studies of a more comprehensive evaluation of outcome, including long-term survival and dynamic changes of functional disabilities, would be warranted to assess the cost-effectiveness of such services.

## Conclusions

In Taiwan, stroke survivors receiving HCBS showed no difference in clinical characteristics from NHIRD nationwide. For them, in addition to the risk factors of comorbidity and stroke severity, both severe functional impairments and cognitive impairments were found as important factors for healthcare utilizations including readmissions and re-emergency. These results regarding reserving functional abilities among stroke survivors were reckoned to be useful information for the ongoing LTC policy reform and the burden of disease for the aged society in Taiwan.

## Data Availability Statement

The raw data supporting the conclusions of this article will be made available by the authors, without undue reservation.

## Ethics Statement

The studies involving human participants were reviewed and approved by Institution Review Board (No: A-ER-106-183). The patients/participants provided their written informed consent to participate in this study.

## Author Contributions

L-FL: study design, resource gathering, study implementation, and writing up of the manuscript. W-MW: data cleaning and statistical analyses. J-DW: consultation of the research method and resource gathering. All authors contributed to the article and approved the submitted version.

## Conflict of Interest

The authors declare that the research was conducted in the absence of any commercial or financial relationships that could be construed as a potential conflict of interest.

## Publisher's Note

All claims expressed in this article are solely those of the authors and do not necessarily represent those of their affiliated organizations, or those of the publisher, the editors and the reviewers. Any product that may be evaluated in this article, or claim that may be made by its manufacturer, is not guaranteed or endorsed by the publisher.
